# Soziale Ungleichheit im Zusammenhang mit digitalen Gesundheitsanwendungen: Digitale Spaltungen in den Bereichen Zugang, Nutzung, Wirksamkeit und Privatsphäre

**DOI:** 10.1007/s00103-024-03832-6

**Published:** 2024-01-30

**Authors:** Tilman Brand, Paula Herrera-Espejel, Saskia Muellmann, Rebekka Wiersing, Heide Busse

**Affiliations:** 1 Leibniz-Institut für Präventionsforschung und Epidemiologie – BIPS, Achterstr. 30, 28359 Bremen, Deutschland; 2 Leibniz-WissenschaftsCampus Digital Public Health Bremen, Bremen, Deutschland

**Keywords:** Digitalisierung, Gesundheitliche Chancengleichheit, Digitale Gesundheitskompetenz, Literalität, Prävention, Digitalization, Equity, Digital health literacy, Language skills, Prevention

## Abstract

In Anbetracht der Zunahme an digitalen Gesundheitsangeboten drängt sich die Frage auf, welche Folgen sich daraus für die gesundheitliche Chancengleichheit ergeben. Ziel dieses narrativen Übersichtsbeitrages ist es, das Ausmaß und die zentralen Aspekte der digitalen Spaltungen (Digital Divide) zu diskutieren. Zur Illustration des Ausmaßes der digitalen Spaltungen wird auf Daten der Liter@te-Studie zurückgegriffen, in der Personen mit einer geringen Literalität (geringe Lese- und Schreibkompetenzen) zur Nutzung von digitalen Gesundheitsangeboten und zu ihrer digitalen Gesundheitskompetenz befragt wurden. Die Ergebnisse der Liter@te-Studie werden mit parallel durchgeführten Bevölkerungsumfragen verglichen. In Bezug auf digitale Spaltungen lassen sich 4 Bereiche unterscheiden: Zugang, Nutzung, Wirksamkeit und Schutz der Privatsphäre. In allen 4 Bereichen lassen sich Ungleichheiten beobachten. Während Unterschiede im Zugang bzw. in der materiellen Infrastruktur sowie im Nutzungsverhalten und in der dafür notwendigen Kompetenz bereits in einigen Studien untersucht wurden, fehlt für eine umfassende Bewertung der ungleichen Wirkungen von digitalen Gesundheitsangeboten in verschiedenen Bevölkerungsgruppen noch die Datengrundlage. Digitale Spaltungen im Bereich des Schutzes der Privatsphäre sind bisher noch wenig untersucht. Transparente und verständliche Datenschutzmaßnahmen werden jedoch zweifelsohne eine wichtige Voraussetzung für den flächendeckenden Einsatz von digitalen Gesundheitsangeboten sein. Insgesamt ist neben einer besseren Studienlage auch eine stärkere Einbeziehung von benachteiligten Adressatengruppen in die Entwicklung von digitalen Gesundheitsangeboten notwendig.

## Abstract (en)

Given the massive increase in digital health tools, the question about the impact of these tools on health equity has gained importance. The aim of this narrative review is to discuss the extent and central aspects of the digital divide. To illustrate the extent of the digital divide, we draw on data from the Liter@te study, which interviewed low-literacy individuals regarding their use of digital health tools and their digital health literacy. The results of the Liter@te study are compared with population surveys conducted in parallel. Four areas can be distinguished in relation to digital divides: access, use, effectiveness, and privacy. Inequalities can be observed in all four areas. While differences in access or material infrastructure, as well as in usage patterns and the required literacies, have already been investigated in some studies, the data basis for a comprehensive assessment of the unequal effects of digital health tools in different population groups is still lacking. Digital divides in the area of privacy protection is an emerging field. However, transparent and understandable privacy measures will undoubtedly be an important prerequisite for the widespread use of digital health tools. Overall, in addition to a better data base, involvement of disadvantaged population groups in the development of digital health interventions is necessary.

## Einleitung

Gesundheitliche Ungleichheiten sind Unterschiede in der Gesundheit zwischen Individuen oder Bevölkerungsgruppen, die auf den ungleichen Zugang zu knappen gesellschaftlichen Ressourcen wie Geld, politischer Macht, Wissen oder vorteilhaften sozialen Beziehungen zurückzuführen sind und als ungerecht beurteilt werden [1–3]. Neben den klassischen Faktoren der vertikalen sozialen Schichtung (Einkommen, Bildung, Beruf) sind hier auch sogenannte horizontale Ungleichheitsfaktoren wie Geschlecht, Migrationsstatus und Alter zu berücksichtigen [4, 5]. Die Gesundheitsforschung hat seit einigen Jahrzehnten den Einfluss dieser sozialen Determinanten auf verschiedene gesundheitsbezogene Verhaltensweisen und Gesundheitsoutcomes untersucht [5]. Darüber hinaus hat das Ziel, gesundheitliche Ungleichheiten zu verringern, Eingang in viele nationale und internationale Politikprogramme, wie z. B. die Ziele für nachhaltige Entwicklung, gefunden [6]. In der Forschung zur gesundheitlichen Ungleichheit hat die Frage, welchen Beitrag Public-Health-Interventionen zur Verstärkung oder Verringerung von gesundheitlichen Ungleichheiten leisten können, in den vergangenen Jahren an Aufmerksamkeit gewonnen [7]. Mit der raschen Zunahme digitaler Public-Health-Maßnahmen rückt auch die Frage nach deren potenziellen Ungleichheitseffekten in den Vordergrund.

Digitale Public-Health-Maßnahmen beziehen sich auf die Integration verschiedener Arten digitaler Technologien zur Verbesserung der zentralen Public-Health-Aufgaben, wie Gesundheitsförderung, Surveillance, bevölkerungsweite Präventionskampagnen und epidemiologische Forschung [8]. Zu den häufig verwendeten digitalen Technologien im Gesundheitsbereich gehören Websites (E-Health-Interventionen), Textnachrichten, E‑Mail-Feedback, Erinnerungsnachrichten oder mobile Anwendungen (M-Health-Interventionen). Weitere Einsatzmöglichkeiten digitaler Technologien können Sensoren oder andere virtuelle Hilfstechnologien zur Förderung von körperlicher Aktivität und zur Verringerung des Sturzrisikos bei älteren Erwachsenen sein [9].

Digitale Public-Health-Maßnahmen verfügen aufgrund ihrer möglichen Zugänglichkeit und Reichweite, Personalisierungsfunktionalitäten, der Möglichkeit, jederzeit und in jeder Umgebung mit Nutzer*innen in Kontakt zu treten, und ihrer geringen ökonomischen Grenzkosten über erhebliche Potenziale [10]. Auf der anderen Seite durchdringt die Digitalisierung alle Ebenen der Determinanten gesundheitlicher Ungleichheit, wie Jahnel et al. in Anlehnung an das sozialökologische Modell von Dahlgren und Whitehead darlegen [11]. Dies unterstreicht, dass trotz der potenziellen Vorteile der Digitalisierung die Gefahr besteht, dass gesundheitliche Ungleichheiten aufrechterhalten oder sogar verschärft werden.

Seit geraumer Zeit wird unter dem Stichwort „Digital Divide“ (digitale Spaltung) die soziale Ungleichheit im Zugang zu digitalen Angeboten und bei deren Nutzung diskutiert [12, 13]. Basierend auf der aktuellen Forschungsliteratur lassen sich digitale Spaltungen in 4, teils aufeinander aufbauenden Bereichen finden: Zugang, Nutzung, Wirksamkeit und Schutz der digitalen Privatsphäre [14–17]. Ein großes Problem in der Erforschung und Bewertung des Ausmaßes der digitalen Spaltungen liegt darin, dass Personengruppen, die über einen erschwerten Zugang zu digitalen Anwendungen verfügen oder diesen gegenüber eine geringe Affinität besitzen, nur selten in nennenswertem Umfang in Studien zu digitalen Public-Health-Maßnahmen einbezogen werden. Eine besondere Personengruppe stellen dabei Menschen mit einer geringen Literalität, d. h. mit geringen Lese- und Schreibkompetenzen, dar. Nach Schätzungen der LEO-Studie (Leben mit geringer Literalität) aus dem Jahr 2018 trifft dies auf ca. 12 % der Erwachsenen in Deutschland zu [18]. Eine geringe Literalität geht häufig mit anderen Benachteiligungsfaktoren wie niedrigen oder gar keinen formalen Bildungsabschlüssen und einem geringen Einkommen einher. Auch Menschen mit einer Migrationsgeschichte weisen nach Ergebnissen der LEO-Studie überproportional häufig eine geringe Literalität auf [18].

Ziel dieses narrativen Übersichtsbeitrages ist es, das Ausmaß und die zentralen Aspekte der digitalen Spaltungen zu diskutieren. Zur Illustration des Ausmaßes der digitalen Spaltungen wird auf Daten der Liter@te-Studie zurückgegriffen, in der Personen mit einer geringen Literalität befragt wurden (siehe Infobox 1). Die Ergebnisse der Liter@te-Studie werden mit parallel durchgeführten Bevölkerungsumfragen verglichen ([19, 20]; siehe Infobox 2).

## Bereiche der digitalen Spaltungen

### Zugang

Der erste Bereich der digitalen Spaltungen, der Zugang, befasst sich mit den Unterschieden zwischen Bevölkerungsgruppen oder Regionen in der Verfügbarkeit von Internetdiensten, mobilen Geräten, Computerhardware oder anderen vernetzten Geräten [12, 21]. Es geht also in erster Linie um die materielle Infrastruktur. Abgebildet wird diese oft mit recht groben Indikatoren wie dem Anteil der Personen mit Internetzugang oder internetfähigen Endgeräten und der Netzabdeckung innerhalb einer Region [22]. Dazu gehören jedoch auch die Möglichkeiten, die Nutzung des Internets über einen längeren Zeitraum aufrechtzuerhalten. Dies erfordert neben der Netzabdeckung auch Software-Abonnements, ein ausreichendes Volumen an mobilen Daten und die Nutzung von nicht veralteten Geräten mit genügend Speicherkapazitäten, aber auch den Zugriff auf Anschlussgeräte wie Bildschirme und Drucker [14].

Ein Ungleichheitsmerkmal, das konsistent mit einem schlechteren Zugang zu digitalen Angeboten assoziiert ist, ist das Alter [23, 24], auch wenn sich die Zahl der Internetnutzenden in der Altersgruppe 60+ in den vergangenen Jahren deutlich gesteigert hat. So zeigen Ergebnisse des Deutschen Alterssurveys aus dem Jahr 2020, dass 86 % der über 60-Jährigen über einen Internetzugang verfügen [25]. Allerdings sind hier deutliche Unterschiede in Bezug auf den Bildungsstand festzustellen. Lediglich 62 % der über 60-Jährigen mit niedrigem Bildungsabschluss verfügen über einen Internetzugang, während es in der hohen Bildungsgruppe 95 % sind [25]. Dies unterstreicht, wie wichtig eine differenzierte und kombinierte Betrachtung von Ungleichheitsfaktoren ist.

Bei Personen mit geringer Literalität zeigen sich deutliche Unterschiede in der Nutzung digitaler Endgeräte für Gesundheitsthemen im Vergleich zu den Ergebnissen der Befragung Digitalisierung und Gesundheit [19]. Während in der Bevölkerungsumfrage 71 % angaben, einen PC oder Laptop zu nutzen, waren dies bei den Personen mit geringer Literalität nur 23 %. Dagegen gaben 79 % an, ein Smartphone oder Tablet in Bezug auf Gesundheitsthemen zu nutzen, während dies in der Allgemeinbevölkerung 70 % waren. Ein Unterschied war auch in der Verfügbarkeit von Aktivitätstrackern und Smartwatches festzustellen (Liter@te-Studie 21 % vs. 35 % in der Bevölkerungsumfrage).

**Figure 1:**
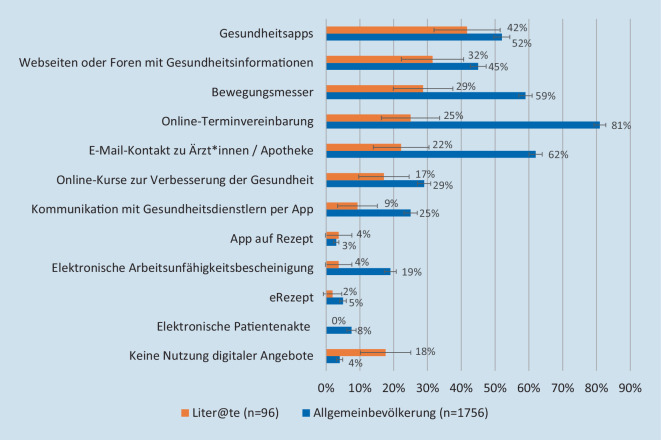

**Figure 2:**
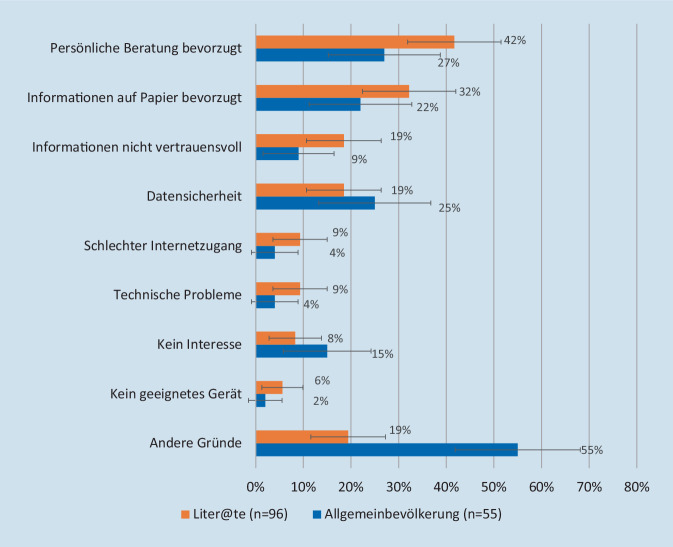

Insgesamt gaben 18 % der Personen mit geringer Literalität an, gar keine digitalen Gesundheitsangebote zu nutzen, während dies in der Befragung der Handelskrankenkasse (hkk) nur 4 % waren (Abb. 1). Auch andere digitale Gesundheitsangebote werden von Personen mit einer geringen Literalität weniger genutzt als von der Allgemeinbevölkerung (z. B. Online-Terminvereinbarung 25 % vs. 81 %, Webseiten oder Foren mit Gesundheitsinformationen 32 % vs. 45 %). Bemerkenswert ist weiterhin, dass unter denjenigen, die angaben, keine digitalen Gesundheitsangebote zu nutzen, in der Liter@te-Studie deutlich mehr Personen Zugangsprobleme berichteten (kein geeignetes Gerät, technische Probleme, schlechte Internetverbindung) als in der Bevölkerungsumfrage (Abb. 2).

### Nutzung

**Figure 3:**
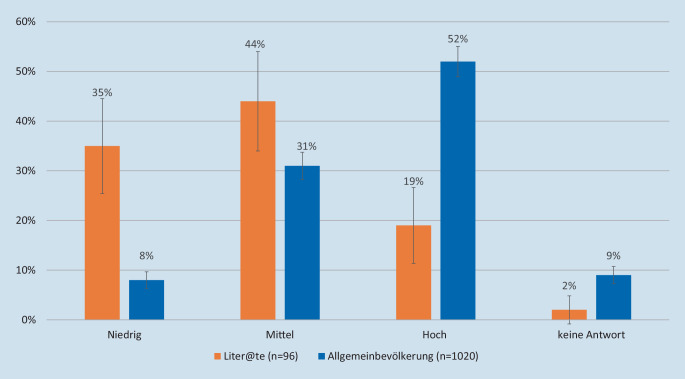

Über den Zugang zur Technologie hinaus wirken sich unterschiedliche Wissensstände und Fähigkeiten auf die individuellen Möglichkeiten aus, digitale Gesundheitsinformationen zu finden und abzurufen, mit dem digitalen Werkzeug zu interagieren und zu kommunizieren oder Inhalte zu erstellen. Daraus ergeben sich unterschiedliche Aneignungsmuster in Bezug auf Nutzungshäufigkeit sowie Such- und Softwarenutzungsgewohnheiten [13]. Der Begriff der digitalen Kompetenz (Digital Literacy) spielt dabei eine zentrale Rolle [26]. Dieser bezieht sich sowohl auf kognitive als auch technische Kompetenzen zum Auffinden, Verstehen, Bewerten und kreativen Nutzen digitaler Werkzeuge und Informationen [27]. Digitale Kompetenz setzt sich aus einer Vielzahl von Fähigkeiten zusammen wie Lese- und Schreibkompetenzen, Medienkompetenz, der Fähigkeit, Informationen richtig verstehen und kritisch hinterfragen zu können, und der Fähigkeit, eine digitale Identität aufzubauen und zu pflegen. Der Begriff der digitalen Gesundheitskompetenz (Digital Health Literacy oder E‑Health Literacy) wird folglich als Fähigkeit definiert, Gesundheitsinformationen aus elektronischen Quellen zu suchen, zu finden, zu verstehen und zu bewerten und die gewonnenen Erkenntnisse bei der Lösung eines Gesundheitsproblems anzuwenden [28]. Wie digitale Gesundheitskompetenz gemessen werden sollte, wird nach wie vor intensiv diskutiert [29]. Ein verbreitetes Instrument hierzu ist die „eHealth Literacy Scale“ (eHEALS; [30]). Bevölkerungsumfragen, die diese Skala benutzen, zeigen laut einer Studie aus dem Jahr 2021 Ungleichheiten in der digitalen Gesundheitskompetenz in Bezug auf Einkommen und Bildung [24]. In dieser Studie liegen die Unterschiede zwischen der niedrigsten und der höchsten Kategorie bei 4 bzw. 5 Skalenpunkten. Während die Bildung und Einkommen eng mit Literalität assoziiert sind, zeigt die Liter@te-Studie, dass der Unterschied in der digitalen Gesundheitskompetenz zwischen Personen mit einer geringen Literalität und der Allgemeinbevölkerung noch größer ist und im Mittel bei 7 Skalenpunkten (entspricht ungefähr 1 Standardabweichung) liegt. Unterteilt man die Skala in die Kategorien niedrig (8–19 Skalenpunkte), mittel (20–29 Skalenpunkte) und hoch (30–40 Skalenpunkte), berichten 35 % in der Liter@te-Studie eine geringe Gesundheitskompetenz im Vergleich zu 8 % in der Bevölkerungsumfrage (Abb. 3).

Um digitale Gesundheitskompetenz nicht zu vereinfacht als individuelle Fähigkeit zu verstehen, ist es wichtig, das Zusammenwirken der materiellen Infrastruktur (Zugang) mit den individuellen Kompetenzen (Nutzung) zu verstehen, wie es transaktionale oder sozialökologische Ansätze nahelegen [29, 31]. Auch Personen mit einer geringen digitalen Gesundheitskompetenz nutzen digitale Angebote. Die entscheidende Frage ist dabei, welches Ausmaß an individuellen Handlungskompetenzen ein digitales Gesundheitsangebot voraussetzt, damit es für die Nutzenden gewinnbringend ist.

### Wirksamkeit

Einzelne digitale Gesundheitsanwendungen vereinen oft Funktionalitäten, die unterschiedliche Schwierigkeitsgrade der Nutzung haben können. Beispielsweise beinhalten Fitness-Apps Schrittzähler, die ohne regelmäßige Interaktion mit den Nutzenden arbeiten, während andere Funktionalitäten (z. B. Ernährungstagebuch) mehr und kompliziertere Interaktionen erfordern. Unterschiede in den Aneignungsmustern, auch wenn sie nur subtil sind, können Personengruppen daran hindern, von einem digitalen Gesundheitsangebot zu profitieren. Obwohl dies ein naheliegendes Risiko für die Vergrößerung von gesundheitlichen Ungleichheiten ist, wird dies in der Forschung und Entwicklung von digitalen Gesundheitsangeboten zu selten berücksichtigt. So kommen Reiners et al. [32] in ihrer Übersichtsarbeit zu dem Schluss, dass bei den meisten digitalen Interventionen für chronische Erkrankungen Unterschiede zwischen den soziodemografischen Merkmalen der Teilnehmenden und ihren digitalen Fähigkeiten nicht berücksichtigt wurden. Die Studien wiesen zudem ein hohes Risiko von Non-Response-Bias auf, was die Verallgemeinerbarkeit ihrer Ergebnisse einschränkte. Abgesehen davon, dass die Studien also relativ blind für ihre differenzielle Wirksamkeit waren, können die Ergebnisse schwerlich für Personengruppen mit eingeschränktem Zugang und eingeschränkten Nutzungskompetenzen extrapoliert werden.

Eine unterschiedliche Wirksamkeit von digitalen Gesundheitsangeboten in verschiedenen Bevölkerungsgruppen (differenzielle Wirksamkeit) ist bisher selten systematisch untersucht worden. Eines der wenigen Beispiele ist die Übersichtsarbeit von Western et al. [33], in die 19 randomisierte kontrollierte Studien im Bereich der Bewegungsförderung eingeschlossen wurden. Die Autor*innen stellen fest, dass digitale Interventionen nur für Personen in einer hohen sozioökonomischen Position wirksam waren und nicht für Personen in einer niedrigen sozioökonomischen Position. Die Übersichtsarbeit von Szinay et al. [34] untersuchte digitale Spaltungen in mehreren Bereichen (Zugang, Nutzung und Wirksamkeit) in Studien, die digitale Interventionen für gewichtsbezogene Verhaltensweisen evaluierten. In den 13 einbezogenen Studien fanden die Autor*innen nur in einer Studie Informationen zu möglichen Ungleichheiten im Zugang und wenig konsistente Ergebnisse zu den Bereichen Nutzung und Wirksamkeit, sodass eine Abschätzung zum Impact des Zusammenwirkens der 3 Bereiche nicht möglich ist. Andere Übersichtsarbeiten zu diesem Thema berichteten ebenfalls über gemischte oder nicht schlüssige Ergebnisse, die den Mangel an verfügbaren Daten zu Ungleichheitsbereichen und deren bisweilen komplexe Interpretation verdeutlichen [35, 36].

In ihrer Gesamtübersicht zu digitalen Spaltungen im Gesundheitsbereich kommen Iyamu et al. [8] zu der Einschätzung, dass sich die meiste Literatur auf die ersten beiden Bereiche der digitalen Spaltung bezieht und Studien zur differenziellen Wirksamkeit weitgehend fehlen. Was allerdings fast noch schwerer wiegt, ist ein Ergebnis, über das Schroeer et al. in ihrer Übersichtsarbeit berichten [37]. Unter den 11 eingeschlossenen Studien war nur eine, die mögliche Ungleichheiten in Zugang, Nutzung oder Wirksamkeit als Limitation diskutierte.

### Wahrung der Privatsphäre

Zusätzlich zu den Bereichen Zugang, Nutzung und Wirksamkeit ist kürzlich vorgeschlagen worden, Ungleichheiten bei der Wahrung der Privatsphäre und der sinnvollen Zustimmung zur Weitergabe personenbezogener Daten als weiteren Bereich digitaler Spaltungen zu betrachten [17]. Der Begriff „Digital Privacy Divide“ beschreibt dabei die Fähigkeit von Personen, ihre Datenschutzrechte im Lichte von modernen Informations- und Kommunikationstechnologienetzwerken, Cloud Computing und Speicherdiensten bewusst wahrzunehmen. Informierte Entscheidungen über die Verwendung der eigenen Daten sind ein Wert an sich. Die Ausnutzung von Unkenntnis über die Datenverwendung oder missbräuchliche Datenverwendung gehören zu den Gefahren der Digitalisierung, zumal Daten bisweilen als „neue Währung“ bezeichnet werden, was den kommerziellen Wert von Nutzer*innen-Daten für Onlinekonzerne verdeutlichen soll.

Auch wenn berücksichtigt werden muss, dass die Forschung zu diesem Bereich noch im Entstehen ist, gibt es erste Hinweise auf Ungleichheiten zwischen Gruppen mit unterschiedlichen Bildungsabschlüssen und digitalen Kompetenzen in Bezug auf das Bewusstsein für Algorithmen, die Wahrnehmung von Datenschutz- und Sicherheitsrisiken und das Vertrauen in die Institutionen, die die eigenen Daten verarbeiten [38]. Das Bewusstsein ist hier nicht das einzige Problem. Die digitalen Spaltungen in diesem Bereich hängen auch mit Ungleichheiten in der materiellen Infrastruktur und den finanziellen Ressourcen zusammen. Beispielsweise gibt es die Möglichkeit zum Kauf sicherer Geräte oder von Programmlizenzen, die das Ausmaß der Datenverfolgung und der Exposition stärker verringern als „kostenlose“ Programmversionen.

Obwohl es in diesem Bereich an Forschung mangelt, kommen Honeyman et al. [38] zu dem Schluss, dass die Wahrnehmung des Datenschutzes ein wesentliches Element bei der Entscheidung für oder gegen die Nutzung eines digitalen Gesundheitsangebotes sein kann. In einer der wenigen Arbeiten, die den Datenschutz in ihrem Scoping-Review erwähnen, untersuchten Mackert et al. [39], wie sich die Wahrnehmung von Datenschutz und Vertrauen auf die Akzeptanz digitaler Gesundheitsangebote in den USA auswirkt. Die Studie ergab, dass ein geringeres Maß an digitaler Gesundheitskompetenz mit einem geringeren Vertrauen in Dienstleistungen von Behörden und Anbietern digitaler Gesundheitsangebote verbunden ist. In der Liter@te-Studie zeigt sich in diesem Zusammenhang ein wichtiger Unterschied zwischen Vertrauen und Datensicherheit. In der Liter@te-Studie erwies sich mangelndes Vertrauen in digitale Informationen als ein gewichtigerer Grund, warum digitale Gesundheitsangebote nicht genutzt wurden, während in der Allgemeinbevölkerung Bedenken bezüglich der Datensicherheit größere Bedeutung hatten (Abb. 2).

Werden die datenschutzrechtlichen Wertvorstellungen verschiedener Bevölkerungsgruppen nicht berücksichtigt oder nicht verständlich dargestellt, kann dies dazu führen, dass die Nutzenden keine angemessenen Bedingungen für eine informierte Zustimmung vorfinden. Im Idealfall umfasst eine solche Aufklärung nicht nur Informationen über die direkte Datennutzung im Rahmen des digitalen Gesundheitsangebots, sondern auch Hinweise zur Gerätesicherheit und zur Vertrauenswürdigkeit der beteiligten Anbieter. Abgesehen von der Nicht-Nutzung kann ein Mangel an Vertrauen in die Datensicherheit auch dazu führen, dass die Nutzenden unvollständige oder bewusste Falschangaben machen, um sich zu schützen.

## Möglichkeiten zum Abbau von digitalen Spaltungen

Auf Grundlage der einbezogenen Forschungsliteratur und der Ergebnisse der Liter@te-Studie lässt sich feststellen, dass Ungleichheiten im Zugang zu digitalen Gesundheitsangeboten und in deren Nutzung bestehen. Weniger deutlich ist die Befundlage in Bezug auf die differenzielle Wirksamkeit sowie das Ausmaß und die Folgen von Ungleichheiten in der Wahrung der Privatsphäre. Als Ungleichheitsfaktoren spielen Einkommen und Bildung eine wichtige Rolle, weil Möglichkeiten, sich kompatible und datensichere Endgeräte, Internetzugänge und Applikationen leisten zu können, und digitale Kompetenzen eng verknüpft sind. Daneben ist das Alter nach wie vor ein wichtiger Ungleichheitsfaktor, auch wenn zeitliche Trends auf eine vermehrte Nutzung von digitalen Angeboten bei älteren Menschen hindeuten [25]. In Bezug auf Herkunft und Sprachfähigkeiten zeigt die Liter@te-Studie sehr deutlich, dass Personen mit einer geringen Literalität digitale Gesundheitsangebote weniger nutzen und sehr häufig eine geringere digitale Gesundheitskompetenz berichten. Hier zeigt sich ein großer Handlungsbedarf, zumal Schätzungen zufolge jede achte Person in Deutschland eine geringe Literalität aufweist [18]. Im Folgenden werden Möglichkeiten diskutiert, wie digitale Spaltungen in den einzelnen Bereichen abgebaut werden können.

Bei Personen mit einer geringen Literalität gehört die Bevorzugung von persönlichen Angeboten zu den wichtigsten Gründen für die Nicht-Nutzung von digitalen Gesundheitsangeboten. Hybride Ansätze, also die Kombination von digitalen und nicht digitalen Elementen, haben das Potenzial, Ungleichheit im Zugang zu reduzieren. Clare [40] hebt in diesem Zusammenhang die Wichtigkeit hervor, bei der Entwicklung und Kombination von digitalen und physischen Interventionselementen die Erfahrungen und Nutzungsgewohnheiten der Adressatengruppen einzubeziehen. Die Liter@te-Studie macht deutlich, dass Personen mit geringer Literalität überwiegend Smartphones oder Tablets und kaum Laptops oder PCs nutzen. Auf der anderen Seite bevorzugen ältere Menschen häufig Laptop und PC [25]. Dies zeigt, dass digitale Gesundheitsangebote für verschiedene Endgeräte verfügbar gemacht werden müssen, um den unterschiedlichen Präferenzen der Adressatengruppen gerecht zu werden. Neben der Optimierung von Inhalten für Smartphones heben Reiners et al. [32] hervor, dass die Inhalte auch im Offline-Modus nutzbar gemacht werden sollten, um Begrenzungen beim Volumen mobiler Daten zu umgehen. Funktionalitäten, wie die einfache Änderung der Schriftgröße sowie Vorlese- und Diktierfunktionen, könnten für Personen mit geringen Lese- und Schreibkompetenzen zu einem Abbau von Zugangsbarrieren beitragen.

Neben der möglichst einfachen und übersichtlichen Gestaltung von digitalen Gesundheitsangeboten ist die gezielte Schulung von individuellen digitalen Gesundheitskompetenzen eine Möglichkeit, um Ungleichheiten in der Nutzung zu verringern. Jenkins et al. [41] betonen, dass man in der Entwicklung von digitalen Gesundheitsangeboten sowohl auf der Adressatenebene als auch hinsichtlich der materiellen Voraussetzungen einen Fokus auf geringe digitale Gesundheitskompetenzen legen sollte. Das bedeutet nicht, dass nur simple digitale Lösungen und einfache Sprache zum Einsatz kommen dürfen. Vielmehr geht es darum, die spezifischen Bedarfe und Nutzungsmuster von Bevölkerungsgruppen mit einer geringen digitalen Gesundheitskompetenz zu kennen und zu berücksichtigen und gegebenenfalls durch (persönliche) Schulungen dafür zu sorgen, dass diese Bevölkerungsgruppe in die Lage versetzt wird, das digitale Gesundheitsangebot gewinnbringend für sich zu nutzen [26]. Als nützlicher Ansatz, um herauszufinden, welche digitalen gesundheitsbezogenen Kompetenzen in den Adressatengruppen vorhanden sind und wie digitale Interventionen an diese Kompetenzen anzupassen sind, kann auf das „eHealth Literacy Framework“ verwiesen werden [42].

Die bisherige Literatur zu Ungleichheiten in der Wirksamkeit von digitalen Gesundheitsangeboten offenbart einen Mangel an Aufmerksamkeit für dieses Thema. Mögliche Ungleichheitseffekte werden oftmals nicht reflektiert, geschweige denn systematisch untersucht. Dies ist zum Teil verständlich, da es sich bei digitalen Gesundheitsangeboten um ein vergleichsweise neues Feld mit einer hohen Dynamik handelt. Es ist deshalb nicht auszuschließen, dass sich Zugangsbarrieren und mangelnde Vertrautheit mit digitalen Anwendungen in einzelnen Bevölkerungsgruppen in den kommenden Jahren deutlich reduzieren, wie die Zunahme der Nutzung von digitalen Angeboten im Alter zeigt. Trotzdem ist ein Mangel an Nutzer*innen-Zentrierung in der Entwicklung von digitalen Gesundheitsangeboten von verschiedenen Autor*innen kritisiert worden [26, 43, 44]. Nutzer*innen-Orientierung beinhaltet die systematische Einbeziehung der Adressatengruppen in den Entwicklungsprozess eines digitalen Gesundheitsangebotes. Partizipative Methoden wie Co-Design oder Co-Kreation können hierzu verwendet werden. Zu beachten gilt es allerdings, dass der Erfolg dieser Methoden sehr stark von deren Umsetzungsqualität abhängt [45]. Weiterhin muss berücksichtigt werden, dass nicht nur digital affine Personen in den Entwicklungsprozess und die Erprobung einbezogen werden, sondern tatsächlich Personen aus benachteiligten Bevölkerungsgruppen mit einer geringen digitalen Gesundheitskompetenz [46].

Die Auswirkungen von Ungleichheiten im Bereich Wahrung der Privatsphäre sind noch nicht hinreichend erforscht ebenso wie die Möglichkeiten, diesen zu begegnen. Aus der Liter@te-Studie wurde ersichtlich, dass Personen mit einer geringen Literalität häufiger eine Verunsicherung berichten, welchen digitalen Informationen sie vertrauen können. Ein Mehr an Informationen durch permanente Zustimmungsabfragen zu Cookies oder Datenschutzbedingungen, wie es seit der Umsetzung der Datenschutzgrundverordnung zu beobachten ist, wird diese Verunsicherung vermutlich eher erhöhen. Nebecker et al. [47] fordern in diesem Zusammenhang mehr Pilotstudien, um herauszufinden, wie eine angemessene Kommunikation über Datenschutz im Rahmen von digitalen Gesundheitsangeboten gestaltet werden kann, damit sie von Personen mit geringen digitalen Gesundheitskompetenzen verstanden werden kann und bestehende Befürchtungen adressiert.

## Fazit

In diesem Betrag wurden Ungleichheiten in den 4 Bereichen der digitalen Spaltungen beschrieben (Zugang, Nutzung, Wirksamkeit und Schutz der Privatsphäre) und anhand von Ergebnissen der Liter@te-Studie illustriert. In den Bereichen Zugang und Nutzung sind deutliche Unterschiede bei verschiedenen Ungleichheitsfaktoren wie Bildung, Einkommen, Alter und Literalität nachweisbar. In den Bereichen Wirksamkeit und Schutz der Privatsphäre gibt es noch erhebliche Forschungslücken. Deshalb ist eine umfassende Bewertung von Ungleichheitseffekten einzelner Formen digitaler Gesundheitsangebote über alle 4 Bereiche hinweg noch nicht möglich. Zukünftige Studien zu digitalen Gesundheitsangeboten sollten möglichst inklusiv angelegt sein, damit auch Bevölkerungsgruppen mit großen Zugangsbarrieren und einer geringen digitalen Gesundheitskompetenz (z. B. Personen mit einer geringen Literalität) angemessen repräsentiert sind. Nutzer*innenzentrierte Ansätze, wie Co-Design und Co-Kreation stellen eine Möglichkeit zur proaktiven Verhinderung von Ungleichheitseffekten dar. Wichtig ist allerdings, dass ein angemessener Einbezug von Personen mit geringen digitalen Gesundheitskompetenzen stattfindet. Weiterhin ist die Entwicklung von verständlichen und vertrauensfördernden Datenschutzerklärungen für digitale Gesundheitsangebote eine Aufgabe für zukünftige Forschung.

### Infobox 1 Die Liter@te-Studie

In der Liter@te-Studie wurden erwachsene Personen mit einer geringen Literalität zu ihrem Zugang und ihrer Nutzung von digitalen Gesundheitsangeboten sowie ihrer digitalen Gesundheitskompetenz befragt. Orientiert an dem Vorgehen der LEO-Studie [18] wurden Personen im mittleren Erwachsenenalter (18–64 Jahre) in die Studie eingeschlossen, die maximal das Alpha-Level 3 erreichten. Das bedeutet, dass sie maximal einzelne Sätze lesen oder schreiben konnten sowie im Leseverständnis an kürzeren zusammenhängenden Texten scheiterten. Die Studie wurde im Frühjahr 2023 in Bremen durchgeführt. Die Teilnehmenden wurden über Sprach- und Integrationskurse sowie Alphabetisierungskurse der Volkshochschule rekrutiert. Mit den Teilnehmenden wurden persönliche Interviews durchgeführt. Mehrsprachige Gesundheitsmediator*innen ermöglichten die Durchführung der Interviews in verschiedenen Sprachen. Insgesamt nahmen 96 Personen an der Befragung teil (im Mittel 44 Jahre alt (Spannweite 21–64), 72 % weiblich, 92 % nicht in Deutschland geboren, 44 % ohne Schulabschluss). Neben soziodemografischen Angaben wurden die Teilnehmenden zu digitalen Geräten und Angeboten, die sie in Zusammenhang mit ihrer Gesundheit nutzen, sowie ihrer digitalen Gesundheitskompetenz (eHEALS [30]) befragt. Dieselben Fragen wurden parallel in 2 Bevölkerungsumfragen erhoben ([19, 20]; siehe Infobox 2).

### Infobox 2 Bevölkerungsumfragen zu „Digitalisierung und Gesundheit“ [19] und „Digitalisierung des Gesundheitswesens“ [20]

**Befragung „Digitalisierung und Gesundheit“**

Im November 2022 wurde eine bundesweite Befragung zum Thema „Digitalisierung und Gesundheit“ durchgeführt [19]. Insgesamt wurden 1020 Internetnutzende ab 18 Jahren (im Mittel 56 Jahre alt, 53 % männlich; Bildungsabschlüsse: 12 % niedrig, 50 % mittel, 38 % hoch) mit Wohnsitz in Deutschland mittels computergestützter Telefoninterviews zu ihrer Einstellung gegenüber digitalen Technologien im Gesundheitskontext und zur Nutzung dieser Technologien befragt. Die digitale Gesundheitskompetenz der Teilnehmenden wurde mit der „eHealth Literacy Scale“ (eHEALS) erfasst [30]. In dem vorliegenden Beitrag werden die Ergebnisse zur Frage, welche digitalen Endgeräte für Gesundheitsangelegenheiten genutzt werden, und zur digitalen Gesundheitskompetenz mit den Ergebnissen der Liter@te-Studie verglichen.

**Online-Befragung von Versicherten der Handelskrankenkasse (hkk) zum Thema „Digitalisierung des Gesundheitswesens**“

Im Februar 2023 nahmen insgesamt 1839 Versicherte der hkk zwischen 18 und 80 Jahren an einer Online-Befragung zum Thema „Digitalisierung des Gesundheitswesens“ teil [20]. 43 % der Teilnehmenden waren männlich, das mittlere Alter lag bei 44 Jahren (Spannweite 18–80), 87 % waren in Deutschland geboren worden, 28 % schätzten ihren sozioökonomischen Status als niedrig ein (41 % mittel, 25 % hoch). Die Teilnehmenden wurden u. a. gefragt, welche digitalen Gesundheitsangebote sie nutzen. Bei denjenigen, die keine Angebote nutzten, wurde nach den Gründen für die Nicht-Nutzung gefragt. Diese Ergebnisse wurden in dem vorliegenden Beitrag mit der Liter@te-Studie verglichen.
